# 

**Published:** 1892-09

**Authors:** 


WHOLE NUMBER, CCCLXXII.

New Series.

September, 1892.

VOL. XXXII. No. 2.

### BUFFALO MEDICAL AND SURGICAL JOURNAL.

Established 1845 by AUSTIN FLINT, M. D.

**Editors:**

THOS. LOTHROP, M. D. WM. WARREN POTTER, M. D.

**Associate Editors:**

WILLIAM C. KRAUSS, M. D. JAMES WRIGHT PUTNAM, M. D.

JOHN A. MILLER, PH. D. DELANCEY ROCHESTER, M. D.

JOHN PARMENTER, M. D.

SCRIPTA FERUNT ANNOS

EUROPEAN AGENCY,
TRÜBNER & CO.,
57 AND 59 LUDGATE HILL, LONDON, E. C.

BUFFALO:
1892.

FOREIGN SUBSCRIPTION, $2.50

Entered at the Post-Office at Buffalo, N. Y., as Second-class Mail Matter.

*Times Printing House, Buffalo, N. Y.*

Table of Contents on Page V.

